# Predictors of Recurrent Acute Chest Syndrome in Pediatric Sickle Cell Disease: A Retrospective Case-Control Study

**DOI:** 10.3390/children9060894

**Published:** 2022-06-15

**Authors:** Abdullah A. Yousef, Hwazen A. Shash, Ali N. Almajid, Ammar A. Binammar, Hamza Ali Almusabeh, Hassan M. Alshaqaq, Mohammad H. Al-Qahtani, Waleed H. Albuali

**Affiliations:** 1 Department of Pediatrics, King Fahad Hospital of the University, Al-Khobar 31952, Saudi Arabia; hashash@iau.edu.sa (H.A.S.); mhqahtani@iau.edu.sa (M.H.A.-Q.); wbuali@iau.edu.sa (W.H.A.); 2 College of Medicine, Imam Abdulrahman Bin Faisal University, Dammam 34221, Saudi Arabia; almajidai.1418@gmail.com (A.N.A.); ammar.a.binammar@gmail.com (A.A.B.); hahm129@hotmail.com (H.A.A.); hassan.m.alshaqaq@gmail.com (H.M.A.)

**Keywords:** acute chest syndrome, sickle cell disease, pediatrics, pulmonary complications, hemoglobinopathy, anemia, Saudi Arabia

## Abstract

Acute chest syndrome (ACS) is a common cause of death in sickle cell disease (SCD) patients. Multiple studies investigated the risk factors of developing ACS; however, predictors of recurrent ACS episodes have not been thoroughly investigated. We aim to examine the clinical and laboratory predictors of recurrent ACS in pediatric patients with SCD. A retrospective case-control study included pediatric patients with SCD (˂14 years) admitted with ACS or developed ACS during admission for another indication. Patients were classified into recurrent ACS episodes (≥2 episodes) and a single ACS episode groups. Ninety-one ACS episodes (42 patients) were included, with a mean age at diagnosis of 7.18 ± 3.38 years. Twenty-two (52.4%) patients were male, and twenty-five (59.5%) patients had recurrent ACS. Younger age at first ACS was significantly associated with recurrence (*p* = 0.003), with an optimal cutoff at 7.5 years (area under the receiver operating characteristic curve [AUROC] = 0.833; *p* < 0.001). Higher SCD-related hospitalizations were significantly associated with recurrence (*p* = 0.038). Higher mean values of baseline white blood count (WBC) (*p* = 0.009), mean corpuscular volume (MCV) (*p* = 0.011), and reticulocyte (*p* = 0.036) were significantly associated with recurrence. Contrarily, lower baseline hematocrit values (*p* = 0.016) were significantly associated with recurrence. The ACS frequencies were significantly lower after hydroxyurea (*p* = 0.021). The odds of ACS recurrence increased with a positive C-reactive protein (CRP) at admission (*p* = 0.006). In conclusion, several baseline and admission laboratory data showed significant associations with recurrence. Hydroxyurea therapy demonstrated reduced ACS episodes.

## 1. Introduction

The most common inherited hemoglobin disorder worldwide is sickle cell disease (SCD) [1]. It is a common condition in the Kingdom of Saudi Arabia (KSA), and most cases are found in the eastern and southwestern provinces [2]. Among SCD patients, acute chest syndrome (ACS) is considered one of the most common causes of death and the second most common cause of hospitalizations following a vaso-occlusive crisis (VOC) [3,4].

ACS is considered a complex and multifactorial etiology [5]. A large multi-center prospective study of 671 ACS episodes reported undetermined etiology in 50% of the cases [5]. The presence of new lung infiltration on chest imaging with either respiratory symptoms or fever describes the definition of ACS [6].

Previous studies have identified several ACS risk factors, including younger age, male sex, chronic respiratory diseases, and hospitalization during certain seasons [7,8,9]. Moreover, it has been found that abdominal surgery increases ACS risk [10]. Buchanan et al. and Kopecky et al. found that opioids in children with painful crises increased the odds of developing ACS [11,12].

Multiple studies were conducted to evaluate the risk factors for developing ACS; however, only a few studies were published regarding the risk factors for recurrent ACS. Patterson et al. found that certain SCD genotypes are associated with a higher risk of developing recurrent ACS episodes [8]. Of these genotypes, patients with hemoglobin HbSS and HbSβ0 thalassemia carry an increased risk of developing recurrent ACS than patients with HbSC and HbSβ+ thalassemia genotypes [8]. Moreover, the study has demonstrated additional risk factors, including a history of asthma or shortness of breath, high platelet count, an extended hospital length of stay (LOS) in the initial hospitalization, ≥2 ACS attacks, and children younger than four years [8]. It has been found that a single ACS episode in early life predicts future attacks of ACS [9]. Furthermore, acute and chronic transfusion therapy was found to be effective in preventing recurrent ACS episodes [13,14].

Given the scarcity of evidence on identifying patients susceptible to recurrent ACS attacks, the main aim of this study was to identify the clinical and laboratory factors that can predict the development of recurrent ACS episodes.

## 2. Materials and Methods

### 2.1. Objectives

The objectives of this study were: (1) to define the clinical features and laboratory values that can predict the development of recurrent ACS episodes; (2) to examine the prediction accuracy of the recurrent ACS predictors; (3) to evaluate the effect of hydroxyurea on the recurrence of ACS episodes.

### 2.2. Study Design and Setting

This was a single-center, retrospective case-control study. It was conducted at King Fahad Hospital of the University (KFHU), Al-Khobar, KSA. The study’s ethical approval was obtained from the ethics committee of the institutional review board (IRB) at Imam Abdulrahman Bin Faisal University.

### 2.3. Patients’ Recruitment

All electronic health records of pediatric patients (˂14 years) labeled with ACS in the period from January 2002 to December 2020 were eligible for screening. We included pediatric patients (˂14 years) who were diagnosed with SCD using hemoglobin electrophoresis and either admitted with ACS or developed ACS during admission for another reason. In contrast, patients with unavailable hemoglobin electrophoresis, uncertain diagnosis of ACS, or unavailable chest X-ray were excluded.

### 2.4. Study Definitions

ACS was defined as the presence of new pulmonary infiltrates on the chest radiography of SCD patients, accompanied by any respiratory symptoms (cough, tachypnoea, wheezing, or shortness of breath), chest pain, or fever of more than 38.5 °C [6]. The ACS diagnosis was further confirmed by a chart review of chest X-ray and clinical features, in which it was reviewed independently by two senior pediatric consultants. Recurrent ACS episodes were defined as having more than one ACS episode, with a minimum of 1 month apart. The patients were categorized into a control group defined as a single ACS (1 episode) and a case group defined as recurrent ACS (≥2 ACS episodes). On the other hand, VOC was defined as new-onset pain in the extremities, back, abdomen, chest, or head that lasted for more than four hours and was not explained by any other etiology other than VOC and required therapy with ketorolac or parenteral opioids in a medical setting [6].

### 2.5. Institutional Guidelines and Protocols

Hydroxyurea therapy was not officially approved by the food and drug administration (FDA) for use in pediatric patients with SCD until December 2017 [15]. Before that date, the patients on hydroxyurea therapy were prescribed according to specific indications by the treating pediatric hematologists. On the other hand, a C-reactive protein (CRP) qualitative test was performed for episodes before 2013, whereas the CRP quantitative test was introduced into the hospital laboratory and available for ACS episodes encountered after 2013. Thus, CRP quantitative findings were converted into positive and negative variables based on the laboratory reference to unify the data measure.

### 2.6. Data Collection

An excel spreadsheet with a predetermined set of variables was adapted based on an extensive literature review. The data were collected by four independent collectors using electronic medical records. Furthermore, to ensure the optimal accuracy of the data collection, it was reviewed by two independent senior pediatricians.

The extraction sheet contains three main domains: baseline data, ACS episodes data, and outcome data. Baseline data was defined as data collected from out-patient clinic visits for one year before the first ACS episode, which included data about patients’ demographics, SCD genotype, age of SCD diagnosis, history of SCD-related adverse events, comorbidities, medications used, and baseline laboratory findings. Information for each ACS episode included season, symptoms at the time of hospital presentation, use of hydroxyurea therapy before the episode, vital signs, physical examination, radiological findings, and laboratory data at several time intervals (at admission, at ACS diagnosis, at the pediatric intensive care unit (PICU) admission if deteriorated, and 24 h before discharge). In addition, during each ACS episode, management data was collected (fluids, analgesics, antibiotics, bronchodilators, maximum oxygen required, transfusion, and chest physiotherapy). Hospital outcome data consisted of PICU admission, need for endotracheal intubation, hospital length of stay (LOS), PICU LOS, and mortality.

### 2.7. Statistical Analysis

The statistical analysis was conducted by the IBM Statistic Package of Social Sciences (SPSS) version 26 (IBM, Armonk, NY, USA). All non-primary missing data were handled by exclusion (pairwise deletion). Data normality was tested using the Kolmogorov-Smirnov test, in which a *p*-value < 0.05 was considered skewed data. Categorical data were presented as frequencies and percentages. In contrast, continuous data were presented as mean and standard deviation (SD) or median and 25th–75th interquartile range (IQR), as appropriate.

Categorical data were compared using chi-square or Fisher’s exact test. On the other hand, continuous data were compared using the independent sample Student’s *t*-test, the Mann-Whitney *U* test, or Wilcoxon Rank-Sum Test as appropriate to the underlying data. A cutoff *p*-value (two-tailed) of 0.05 was considered statistically significant. A univariable binary logistic regression was performed on variables that showed statistically significant differences (*p*-value ≤ 0.05). Unadjusted odds ratio (UOR), 95% confidence interval (CI), and the *p*-value were reported for the regression analysis.

The prediction performance of the significant continuous variables predicting recurrent ACS over the range of possible cutoff values was evaluated using the receiver operating characteristics (ROC) to calculate the area under the curve (AUC) using a nonparametric distribution assumption. We reported the area under the receiver operating characteristic curve (AUROC), 95% CI, and the *p*-value for this predictive model. Optimal cutoff thresholds were chosen based on the Closest to (0, 1) Criteria (balancing the sensitivity and specificity). After identifying the optimal cutoff values, we assessed the prediction accuracy for the identified optimal cutoff points using sensitivity, specificity, positive predictive value (PPV), negative predictive value (NPV), positive likelihood ratio (LR+), and negative likelihood ratio (LR−).

## 3. Results

**Figure 1:**
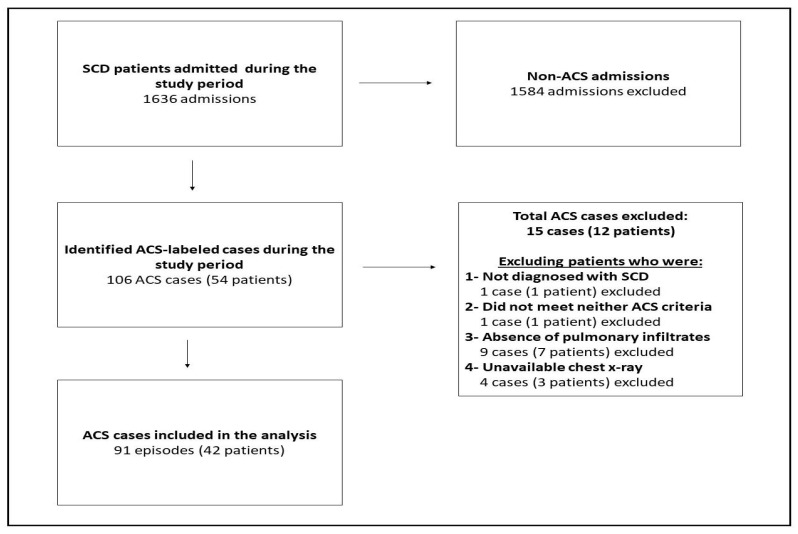
Flow chart showing the screening, inclusion, and exclusion processes. SCD, Sickle Cell Disease; ACS, Acute Chest Syndrome.

The analysis included 91 ACS episodes with a total of 42 patients (Figure 1). Of the 91 episodes, 17 episodes were non-recurrent (single ACS event per patient). The median of ACS episodes in the recurrent group was 2 (IQR, 1–3). Of the 42 patients, 22 (52.4%) were males (Table 1). No significant difference was observed between genotypes and the development of recurrent ACS.

At diagnosis of ACS episodes (*n* = 91), the overall mean age was 7.18 ± 3.38 years. Patients with recurrent ACS episodes showed a significantly lower mean of age for overall episodes (6.76 ± 3.39 vs. 9 ± 2.76 years; *p* = 0.013) (Table 2). The mean age at the first ACS episode was 6.62 ± 3.38 years. Patients with recurrent ACS had a younger age at the first ACS episode (5.16 ± 3.13) than those with a single ACS episode (8.67 ± 2.54), with a statistically significant difference (*p* < 0.001). There was no statistically significant difference between male and female patients regarding ACS recurrence (*p* = 0.491). Male patients were more likely to present with their first ACS at a younger age than female patients (6.42 ± 4.07 vs. 7.25 ± 2.81 years); however, this difference did not meet the statistical significance (*p* = 0.441).

The absolute number of sickle cell disease-related hospitalizations per year was higher among patients with recurrent ACS episodes (4 [IQR, 2–7] vs. 2 [IQR, 1–4]; *p* = 0.026). Although higher frequencies of comorbidities were observed in the recurrent ACS group, these differences did not reach the statistical significance level. Remarkably, 8/9 patients with more than three ACS episodes had at least one comorbidity.

At baseline, higher mean values were found to be associated with the recurrent ACS group, including the white blood count (WBC) with a mean of 16.87 ± 7.24 vs. 11.13 ± 3.73 (*p* = 0.013), mean corpuscular volume (MCV) with a mean of 81.89 ± 7.64 vs. 70.91 ± 15.47 (*p* = 0.017), reticulocyte count with a mean of 11.21 ± 4.66 vs. 7.81 ± 3.95 (*p* = 0.031), and total and direct bilirubin (3.19 vs. 1.25 and 0.4 vs. 0.25, *p* = 0.011 and *p* = 0.008, respectively) (Table 3). On the other hand, lower mean values were found to be associated with recurrent ACS, including the red blood cell (RBC) count with a mean of 2.91 ± 0.39 vs. 3.52 ± 0.89 (*p* = 0.024), and hematocrit with a mean of 23.45 ± 1.83 vs. 25.78 ± 3.48 (*p* = 0.026). A comparison between hematological variables in light of SCD genotypes was performed and is presented in the (Supplementary Material Table S1).

Comparing laboratory variables at different time intervals between the two study groups showed that neutrophils at time of ACS diagnosis, lymphocytes at PICU admission, RBC 24 h before discharge, MCV at admission, MCV at ACS diagnosis, MCV 24 h before discharge, mean corpuscular hemoglobin (MCH) at admission, MCH at ACS diagnosis, and positive qualitative CRP at admission had a statistically significant difference between the two groups (Supplementary Material Table S2).

**Figure 2:**
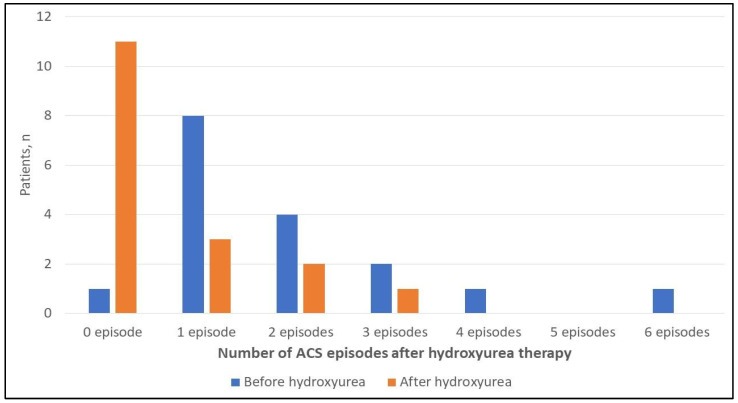
Clustered bar chart illustrating the difference in ACS episodes frequency before and after hydroxyurea therapy [17 patients]. ACS, Acute Chest Syndrome.

In patients who were started on hydroxyurea, the number of ACS episodes before and after initiating hydroxyurea were significantly different (*p* = 0.021), with a lower median observed after using hydroxyurea (0 [IQR, 0–1] vs. 1 [IQR, 1–2.5]; Table 4 and Figure 2). The median hospital LOS among all episodes was eight days (IQR, 5–10.25). Moreover, the median PICU LOS among all episodes was 4 (IQR, 3–5.5). The survival rate was 100%.

In univariable logistic regression, several risk factors were significantly related to the development of recurrent ACS episodes (Table 5). Notably, for every 1-year increase in age at the time of the first ACS diagnosis, there was a 32.8% decrease in the odds of having a recurrent ACS (*p* = 0.003). Moreover, the baseline RBC value showed a 79% decrease in the odds of having recurrent ACS for every one-unit increase (*p* = 0.016). The odds of having recurrent ACS were dramatically increased by an odds ratio of 9.333 with a positive CRP at the time of admission (UOR, 9.333; 95% CI, 1.919–45.386; *p* = 0.006); however, the wide CI possess very serious risk of imprecision that is in large extent due to the small sample size of the control group. Despite the significant reduction in ACS frequencies after using hydroxyurea demonstrated by the Wilcoxon Rank-Sum Test for the group of patients on hydroxyurea therapy (*n* = 17), the logistic analysis failed to achieve statistical significance when hydroxyurea was examined among the 91 ACS episodes (UOD, 0.465; 95% CI, 0.138–1.562; *p* = 0.215); nevertheless, the estimate is affected by imprecision (very wide CI) due to low number of patients on hydroxyurea to estimate an effect reliably.

In assessing the predictive ability to discriminate recurrent ACS from single ACS patients, ROC analysis indicated an excellent discrimination ability of age at the time of first ACS diagnosis (AUROC = 0.833; *p* < 0.001). The optimal cutoff point for age at ACS diagnosis was ≤7.5 years, which showed a sensitivity of 84% and specificity of 76.5% in identifying patients with recurrent ACS. Furthermore, the likelihood ratio positive of age at the time of ACS was 3.57, indicating that patients who presented with their first ACS before 7.5 years of age had an approximate +20% change in the probability of recurrent ACS. On the other hand, patients who presented after 7.5 years of age had an approximate -30% change in ACS recurrence probability (LR-, 0.21) (Supplementary Material Tables S3 and S4).

The predictive performance of the baseline WBC, MCV, hematocrit, and reticulocytes showed a moderate discrimination ability with AUROC of 0.786 (*p* = 0.009), 0.767 (*p* = 0.011), 0.754 (*p* = 0.016), and 0.721 (*p* = 0.036), respectively. In contrast, baseline RBC did not achieve statistical significance (AUROC, 0.7; *p* = 0.058), indicating a poor discrimination ability that was not statistically different from chance alone.

## 4. Discussion

The burden of ACS on pediatric SCD patients combined with the scarcity of evidence is a major challenge to pediatric hematologists. This study demonstrated that early age at first ACS diagnosis and a higher incidence of SCD-related hospitalizations were significant risk factors of recurrent ACS episodes. Moreover, there was a significant association between recurrent ACS episodes and higher mean baseline values of WBC count, reticulocytes count, MCV, and total and direct bilirubin. In contrast, lower baseline values of hematocrit and RBC count were associated with an increased risk of recurrent ACS episodes. Notably, a positive CRP test at admission was markedly associated with recurrent ACS episodes. Although CRP demonstrated statistical association, the clinical utility is questionable in this population.

Our study showed that the earlier the first ACS episode, the higher the risk for developing recurrent ACS episodes, specifically for patients who experience their first ACS below 7.5 years of age. Similarly, Patterson et al. and DeBaun et al. found that an early first ACS episode was significantly associated with the development of future ACS episodes; however, they reported a different cutoff value (<4 years) [8,9].

Despite the higher frequencies of doctor-diagnosed asthma in the recurrent ACS group (9 patients) than in the single ACS (2 patients) group, a significant difference was not demonstrated in our cohort. In contrast, Knight-Madden et al. demonstrated that doctor-diagnosed asthma (reported by parents) resulted in a 6-fold increase in the odds of having ACS recurrence [16]. Additionally, Boyd et al. reported that SCD patients with asthma are at higher risk of developing ACS episodes; however, they could not determine whether SCD patients with comorbid asthma is associated with more frequent ACS episodes [17].

Paterson et al. found that HbSC genotype, Sβ0 thalassemia genotype, history of asthma, shortness of breath at admission, increased platelet counts at the initial hospitalization, and increased hospital LOS was significantly associated with a higher risk of having recurrent ACS episodes [8]. Moreover, the study by Paterson et al. demonstrated that being a male is a risk of developing recurrent ACS episodes, while DeBaun et al. noticed that females were at a higher risk [8,9]. On the contrary, none of these factors were significant in our analysis.

The prevention of recurrent ACS has been the purpose of multiple studies. Our study findings demonstrated that hydroxyurea therapy might lower the absolute count of ACS episodes following its use. This was evident with a statistically significant (*p* = 0.021) reduction of ACS episode frequencies after starting hydroxyurea therapy. Our results are consistent with the Pediatric Hydroxyurea Phase 3 Clinical Trial (BABY HUG) in sickle cell syndromes results, which showed a significant decrease in ACS incidence from 14.6 patients per 100 on placebo compared to 4.2 patients per 100 on hydroxyurea therapy [18]. However, we did not observe a statistically significant difference when examining hydroxyurea’s use across all ACS episodes as a possible protective factor. Nevertheless, a few issues need to be considered before making a solid conclusion. First, the small sample of patients on hydroxyurea limits the detection of the effect size and the significant difference. Second, some patients have already encountered one or more episodes before starting hydroxyurea therapy, classifying them among the recurrent ACS group. Third, the use of hydroxyurea therapy was not a possible choice for all the included patients, of which 19 patients (45%) were already transferred to adult care before the commencement of the institutional hydroxyurea guidelines, where the use of hydroxyurea therapy was expanded considerably. Similarly, Patterson et al. performed a similar study in children prior to the approval of hydroxyurea. They found no significant association between hydroxyurea use and multiple ACS episodes in a sample size of patients on hydroxyurea double that in our study (*n* = 37) [8]. Our study suggested the importance of starting hydroxyurea early to prevent recurrent ACS, and further study is needed in our population, particularly on the Arab-Indian subtype of sickle cell anemia in Saudi Arabia [2].

Chronic transfusion therapy is a commonly used modality of treatment in patients with sickle cell anemia, particularly in primary and secondary stroke prevention [19]. Chronic transfusion demonstrated a marked reduction in the number of hospitalizations due to ACS [14,20]. Though the studies indicate that chronic blood transfusion may reduce the incidence of ACS; there is currently no reliable evidence to support or negate its use [21]. A Cochrane review concluded that treatment should be based on clinical experience, surrounding circumstances, and patient population characteristics. [21]. The newer agents available for the treatment of sickle cell anemia include L-glutamine, crizanlizumab, and voxelotor. These drugs are beneficial in increasing the hemoglobin concentration or decreasing the frequency of VOCs, which may indirectly reduce the frequency of ACS [22]. However, there has been no proven benefit reported that these drugs may directly reduce the frequency of ACS [22]. Hematopoietic stem cell transplantation (HSCT) provides curative therapy for children with SCD, as it eradicates acute complications and prevents further end-organ damage [23]. Due to the complications associated with HSCT, recurrent ACS is an indication for referral only if recurrence occurs despite optimal dosing of hydroxyurea [23]. Despite the advances in the treatment of SCD, hydroxyurea remains the standard of care for patients with recurrent ACS.

The current study findings should be further investigated in future studies with a larger sample size and a superior study design to confirm replicability. Furthermore, these findings should be studied regarding their effects on patients’ management and overall outcomes. This research has identified several predictors of recurrent ACS with prediction accuracy ranging from excellent to moderate; nevertheless, further research needs to combine these predictors to develop clinical decision-making criteria that can have the highest diagnostic accuracy in discriminating patients predisposed to recurrent ACS.

## 5. Strengths and Limitations

This study possesses several strengthening points. First, it includes 18 years of experience in a tertiary teaching hospital with rigorous inclusion and exclusion criteria. Second, this is the first study in Saudi Arabia to study the overall discrimination accuracy of clinical and laboratory variables toward recurrent ACS over a range of possible thresholds. Third, having independent senior pediatricians review the collected data has ensured further accuracy. Finally, the collected variables were extracted rigorously using a predetermined protocol to minimize extraction errors and missing values.

Despite the above-mentioned strengths, inherent limitations deserve to be considered. First, it was a single-center study with a small sample size; therefore, this study’s findings might have limited generalizability to other healthcare settings. Second, we could not confirm the absence of possible extra ACS episodes that might be admitted to other hospitals. Third, potential unmeasured bias cannot be ruled out. Besides, covariates adjustment for the regression and ROC models to exclude the confounding effects was not possible due to the small size of the two groups, particularly the control group (*n* = 17). Due to the study’s retrospective nature, the collected data is subjected to the primary treating team’s entirety of documentation.

## 6. Conclusions

In conclusion, this study indicates several factors that might predict the recurrence of ACS. The risk factors described are from clinical evaluation and baseline laboratory investigations, which may assist physicians in resource-limited settings in anticipating the recurrence of ACS. These factors include age at the first ACS episode, the time between SCD diagnosis and the first ACS episode, and the number of SCD-related hospitalizations. Furthermore, multiple baseline and admission laboratory values demonstrated significant associations, which might help predict recurrent ACS episodes. These studied clinical and laboratory predictors might assist clinicians in the risk assessment of this vulnerable population. Even with the provided evidence on the predictors and prediction accuracy, future large-scale controlled studies are warranted to confirm the current study’s findings.

## Figures and Tables

**Table 1:** Demographics and baseline characteristics of the included patients [*n* = 42].

Study Variables	Total Population (*n* = 42)	Patients with Single ACS Episode (*n* = 17)	Patients with Recurrent ACS Episodes (*n* = 25)	*p*-Value
Age at the time of SCD Diagnosis, median (IQR) *, months	8 (6–24)	8.5 (5.25–36)	8 (6–24)	0.911 ^a^
Male:Female ratio, *n* (%)	22:20 (52.4%:47.6%)	10:7 (58.8%:41.2%)	12:13 (48%:52%)	0.491 ^b^
Hgb SS, *n* (%)	29 (69%)	12 (70.6%)	17 (68%)	0.859 ^b^
Hgb Sβ0 thalassemia, *n* (%)	9 (21.4%)	3 (17.6%)	6 (24%)	0.490 ^b^
Hgb Sα thalassemia, *n* (%)	4 (9.5%)	2 (11.8%)	2 (8%)	1 ^b^
Age at the time of first ACS diagnosis, mean ± SD	6.62 ± 3.38	8.67 ± 2.54	5.16 ± 3.13	<0.001 **^c^
Number of acute anemia/year, mean ± SD	3.64 ± 1.74	2.88 ± 1.46	4.67 ± 1.63	0.051 ^c^
Number of VOC/year, median (IQR)	2 (1–3)	2 (1.25–3)	2 (1–3)	0.899 ^a^
SCD-related hospitalizations/year, median (IQR)	3 (2–4)	2 (1–4)	4 (2–7)	0.026 **^a^
Comorbidities:				
G6PD deficiency, *n* (%)	9 (21.4%)	4 (23.5%)	5 (20%)	1 ^b^
Asthma, *n* (%)	11 (26.2%)	2 (11.8%)	9 (36%)	0.151 ^b^
CVS diseases, *n* (%) ^d^	2 (4.8%)	0	2 (8%)	0.506 ^b^
Endocrine diseases, *n* (%) ^e^	3 (7.1%)	1 (5.9%)	2 (8%)	1 ^b^
Developmental delay, *n* (%)	2 (4.8%)	0	2 (8%)	0.506 ^b^
OSA, *n* (%)	1 (2.4%)	0	1 (4%)	1 ^b^
Miscellaneous, *n* (%) ^f^	4 (9.52%)	0	4 (9.52%)	0.134 ^b^
≥ 1 comorbidities, *n* (%)	22 (52.4%)	7 (41.2%)	15 (60%)	0.231 ^b^
Medications before ACS episodes:				
Hydroxyurea therapy at baseline, *n* (%)	1 (2.4%)	0	1 (4%)	1 ^b^
Folic acid, *n* (%)	39 (92.9%)	15 (88.2%)	24 (96%)	0.556 ^b^
Penicillin V, *n* (%) *	28 (66.7%)	14 (82.4%)	14 (56%)	0.075 ^b^
Regular transfusion, *n* (%) *	2 (4.8%)	1 (5.9%)	1 (4%)	1 ^b^
Ventolin, *n* (%) *	5 (11.9%)	2 (11.8%)	3 (12%)	1 ^b^
Aspirin, *n* (%) *	1 (2.4%)	0	1 (4%)	1 ^b^
Amlodipine, *n* (%) *	1 (2.4%)	0	1 (4%)	1 ^b^

* Missing details were excluded. ** Significant at *p* ≤ 0.05 level. ^a^ *p*-value was calculated using the Mann-Whitney *U* test. ^b^ *p*-value was calculated using a chi-square test or Fisher’s exact test. ^c^ *p*-value was calculated using the independent Student’s *t*-test. ^d^ CVS diseases: HTN and CHD. ^e^ Endocrine diseases: Type-1 DM, rickets, and SIADH. ^f^ Miscellaneous: Epilepsy, achalasia, autism, and eczema. Abbreviations: ACS, Acute Chest Syndrome; SCD, Sickle Cell Disease; VOC, Vaso-occlusive Crisis; AVN, Avascular Necrosis; G6PD, Glucose-6-phosphate Dehydrogenase; CVS, Cardiovascular System; OSA, Obstructive Sleep Apnea.

**Table 2:** ACS episodes’ characteristics, clinical features, and physical examination (*n* = 91).

Study Variables	Total Population (*n* = 91)	Non-Recurrent ACS (*n* = 17)	Recurrent ACS (*n* = 74)	*p*-Value
Age at time of all ACS episodes’ diagnoses, mean ± SD	7.18 ± 3.38	9 ± 2.76	6.76 ± 3.39	0.013 **^a^
ACS episodes required PICU admission, *n* (%)	9 (9.9%)	2 (11.8%)	7 (9.5%)	0.673 ^c^
Hydroxyurea before each ACS episode, *n* (%)	10 (11%)	0	10 (13.5%)	0.2 ^c^
Clinical features at hospital presentation:				
SOB, *n* (%)	47 (51.6%)	12 (70.6%)	35 (47.3%)	0.083 ^c^
Fever, *n* (%)	64 (70.3%)	11 (64.7%)	53 (71.6%)	0.574 ^c^
Cough, *n* (%)	64 (70.3%)	10 (58.8%)	54 (73%)	0.249 ^c^
Chest pain, *n* (%)	30 (33%)	7 (41.2%)	23 (31.1%)	0.425 ^c^
Extremity pain, *n* (%)	18 (19.8%)	5 (29.4%)	13 (17.6%)	0.314 ^c^
Back pain, *n* (%)	15 (16.5%)	6 (35.3%)	9 (12.2%)	0.031 **^c^
URTI symptoms, *n* (%)	20 (22%)	3 (17.6%)	17 (23%)	0.755 ^c^
GI symptoms, *n* (%)	15 (16.5%)	4 (23.5%)	11 (14.9%)	0.468 ^c^
Others, *n* (%) ^d^	6 (6.6%)	1 (5.9%)	5 (6.8%)	1 ^c^
Vital signs:				
Heart rate, mean ± SD	118.78 ± 22.09 bpm	117.81 ± 25.74 bpm	119.02 ± 21.31 bpm	0.847 ^a^
Respiratory rate, median (IQR)	32 (24–42) bpm	33 (25–42.5) bpm	32 (22–41) bpm	0.679 ^b^
Temperature, mean ± SD	37.91 ± 0.97 °C	37.54 ± 0.94 °C	37.99 ± 0.96 °C	0.105 ^a^
SBP, mean ± SD	104.62 ± 11.42 mmHg	102 ± 7.36 mmHg	105.29 ± 12.21 mmHg	0.323 ^a^
DBP, median (IQR)	59 (55–62) mmHg	61 (58.5–66) mmHg	59 (55–60) mmHg	0.507 ^b^
MAP, mean ± SD	77.08 ± 8.8 mmHg	76 ± 6.44 mmHg	77.36 ± 9.34 mmHg	0.597 ^a^
SpO_2_, median (IQR)	96 (89–99)%	99 (88–99)%	95.5 (89.75–97.25)%	0.471 ^b^
Chest examination:				
Wheezing, *n* (%)	10 (11%)	1 (5.9%)	9 (12.2%)	0.681 ^c^
Crackles, *n* (%)	44 (48.4%)	5 (29.4%)	39 (52.7%)	0.083 ^c^
Reduced breath sound, *n* (%)	33 (36.3%)	8 (47.1%)	25 (33.8%)	0.305 ^c^
Respiratory distress, *n* (%) ^e^	17 (18.7%)	1 (5.9%)	16 (21.6%)	0.179 ^c^

** Significant at *p* ≤ 0.05 level. ^a^ *p*-value was calculated using the independent *t*-test. ^b^ *p*-value was calculated using the Mann-Whitney *U* test. ^c^ *p*-value was calculated using a chi-square test or Fisher’s exact test. ^d^ Others include generalized edema, decreased feeding, and decreased activity, and headache. ^e^ Respiratory distress was defined as the presence of ≥1 of the following: use of accessory muscles, increased work of breathing, intercostal retraction, and grunting. Abbreviations: ACS; Acute Chest Syndrome; SD, Standard Deviation; IQR, Interquartile Range; ER, Emergency Room; PICU, Pediatric Intensive Care Unit; SOB, Shortness of Breath; URTI, Upper Respiratory Tract Infection; GI, Gastrointestinal; SBP, Systolic Blood Pressure; DBP, Diastolic Blood Pressure; MAP, Mean Arterial Pressure; SpO_2_, Oxygen Saturation.

**Table 3:** Baseline laboratory data of the included patients (*n* = 42).

Study Variables	Total Population (*n* = 42)	Patients with Single ACS Episode (*n* = 17)	Patients with Recurrent ACS Episodes (*n* = 25)	*p*-Value
WBC count, mean ± SD, (*n* = 29)	14.1 ± 6.42 k/uL	11.13 ± 3.73 k/uL	16.87 ± 7.24 k/uL	0.013 **^a^
Neutrophils, mean ± SD, (*n* = 23)	40.59 ± 14.56 %	44.11 ± 13.43%	35.11 ± 15.54%	0.155 ^a^
Eosinophils, median (IQR), (*n* = 27)	3 (1.9–4%)	2 (1.1–4.1)%	3 (2.08–4)%	0.581 ^b^
Lymphocytes, mean ± SD, (*n* = 27)	43.69 ± 16.24%	40.98 ± 16%	47.1 ± 16.58%	0.342 ^a^
Hemoglobin, mean ± SD, (*n* = 31)	8.28 ± 0.88 g/dL	8.57 ± 0.9 g/dL	8 ± 0.80 g/dL	0.07 ^a^
MCV, mean ± SD, (*n* = 31)	76.58 ± 13.11 fL	70.91 ± 15.47 fL	81.89 ± 7.64 fL	0.017 **^a^
MCH, mean ± SD, (*n* = 31)	30.13 ± 13.51 pg	29.32 ± 15.32 pg	30.89 ± 12.04 pg	0.751 ^a^
RBC count, mean ± SD, (*n* = 31)	3.2 ± 0.732 Mil/uL	3.52 ± 0.89 Mil/uL	2.91 ± 0.39 Mil/uL	0.024 **^a^
Hematocrit, mean ± SD, (*n* = 31)	24.58 ± 2.95%	25.78 ± 3.48 %	23.45 ± 1.83%	0.026 **^a^
Reticulocyte count, mean ± SD, (*n* = 31)	9.57 ± 4.46%	7.81 ± 3.95 %	11.21 ± 4.66%	0.031 **^a^
Platelets, mean ± SD, (*n* = 30)	410.9 ± 184.15 k/uL	418.4 ± 220.24 k/uL	403.4 ± 147.03 k/uL	0.828 ^a^
BUN, mean ± SD, (*n* = 16)	7.47 ± 2.69 mg/dL	8.38 ± 3.06 mg/dL	6.56 ± 2.06 mg/dL	0.186 ^a^
Creatinine, median (IQR), (*n* = 15)	0.3 (0.3–4) mg/dL	0.4 (0.28–0.4) mg/dL	0.3 (0.3–0.4) mg/dL	0.536 ^b^
Total bilirubin, mean ± SD, (*n* = 12)	2.54 ± 1.61 mg/dL	1.25 ± 0.48 mg/dL	3.19 ± 1.59 mg/dL	0.011 **^a^
Direct bilirubin, median (IQR), (*n* = 12)	0.3 (0.293–0.475) mg/dL	0.25 (0.2–0.3) mg/dL	0.4 (0.3–0.5) mg/dL	0.008 **^b^
AST, median (IQR), (*n* = 12)	48 (41.5–76.75) U/L	66 (29.75–149.5) U/L	45.5 (41.5–58.5) U/L	0.570 ^b^
ALT, median (IQR), (*n* = 12)	29.5 (24.75–44.375) U/L	40 (27.75–96.5) U/L	28.5 (21–32.63) U/L	0.154 ^b^
Alkaline phosphates, mean ± SD, (*n* = 12)	175.5 ± 40.42 U/L	171.5 ± 31.93 U/L	177.5 ± 46 U/L	0.821 ^a^
LDH, mean ± SD, (*n* = 12)	519.5 ± 184.55 U/L	434.5 ± 202.69 U/L	562 ± 172.39 U/L	0.279 ^a^

** Significant at *p* ≤ 0.05 level. ^a^ *p*-value was calculated using the independent Student’s *t*-test. ^b^ *p*-value was calculated using the Mann-Whitney *U* test. Abbreviations: ACS, Acute Chest Syndrome; WBC, White Blood Cells; SD, Standard Deviation; IQR, Interquartile range; MCV, Mean Corpuscular Volume; MCH, Mean Corpuscular Hemoglobin; RBC, Red Blood Cells; BUN, Blood Urea Nitrogen; AST, Aspartate Aminotransferase; ALT, Alanine Aminotransferase; LDH, Lactate Dehydrogenase.

**Table 4:** Clinical outcomes of ACS episodes.

Study Variables	Total Population (*n* = 91)	Single ACS Episode (*n* = 17)	Recurrent ACS Episodes (*n* = 74)	*p*-Value
Hospital LOS, median (IQR) *	8 (5–10.25) (*n* = 86)	9 (6–14) (*n* = 15)	7 (5–10) (*n* = 71)	0.108 ^a^
PICU LOS, median (IQR)	4 (3–5.5) (*n* = 9)	21.5 (12.25–30.75) (*n* = 2)	4 (3–5) (*n* = 7)	0.667 ^a^
Number of ACS episodes before initiating hydroxyurea, median (IQR)	1 (1–2.5) (*n* = 17)	1 (1–1) (*n* = 5)	2 (1–3) (*n* = 12)	0.021 **^b^
Number of ACS episodes after initiating hydroxyurea, median (IQR)	0 (0–1) (*n* = 17)	0 (0–0) (*n* = 5)	0.5 (0–1.75) (*n* = 12)	

* Missing details were excluded. ** Significant at *p* ≤ 0.05 level. ^a^ *p*-value was calculated using the Mann-Whitney *U* test. ^b^ *p*-value was calculated using Wilcoxon signed rank test for the entire population. Abbreviations: ACS, Acute Chest Syndrome; LOS, Length of Stay IQR, Interquartile Range; PICU, Pediatric Intensive Care Unit.

**Table 5:** Univariable binary logistic regression analysis of potential factors associated with developing recurrent ACS.

Study Variables	UOR (95% CI)	*p*-Value
Age at time of first ACS diagnosis (per 1-year increase), (*n* = 42/42)	0.672 (0.515–0.876)	0.003 **
Age at time of all ACS episodes’ diagnoses (per 1-year increase), (*n* = 91/91)	0.805 (0.674–0.963)	0.017 **
SCD-related hospitalizations/year (per 1 hospitalization increase), (*n* = 37/42)	1.639 (1.027–2.616)	0.038 **
Baseline WBC count (per 1-unit increase), (*n* = 29/42)	1.267 (1.027–1.564)	0.028 **
Baseline MCV (per 1-unit increase), (*n* = 31/42)	1.108 (1.010–1.215)	0.031 **
Baseline RBC count (per 1-unit increase), (*n* = 31/42)	0.211(0.049–0.913)	0.037 **
Baseline hematocrit (per 1-unit increase), (*n* = 31/42)	0.714 (0.519–0.983)	0.039 **
Baseline reticulocyte count (per 1-unit increase), (*n* = 31/42)	1.253 (1.005–1.562)	0.045 **
Back pain, (*n* = 91/91)		
- No back pain at presentation	Reference	-
- Back pain at presentation	0.254 (0.075–0.855)	0.027 **
Neutrophil at time of ACS diagnosis (per 1-unit increase), (*n* = 64/91)	0.957 (0.917–0.998)	0.041 **
RBC count 24 h before discharge (per 1-unit increase), (*n* = 32/91)	0.029 (0.002–0.514)	0.016 **
MCV at time of admission (per 1-unit increase), (*n* = 89/91)	1.084 (1.021–1.150)	0.008 **
MCV at time of diagnosis (per 1-unit increase), (*n* = 86/91)	1.087 (1.020–1.157)	0.010 **
MCV 24 h before discharge (per 1-unit increase),(*n* = 32/91)	1.137 (1.005–1.287)	0.042 **
Qualitative CRP at time of admission, (*n* = 74/91):		
- Negative CRP	Reference	-
- Positive CRP	9.333 (1.919–45.386)	0.006 **
Use of NSAIDs, (*n* = 91/91):		
- No NSAIDs	Reference	-
- NSAIDs	0.278 (0.092–0.840)	0.023 **
Use of clarithromycin, (*n* = 91/91):		
- No clarithromycin	Reference	-
- Clarithromycin	0.064 (0.006–0.660)	0.021 **
Use of hydroxyurea therapy, (*n* = 91):		
- No hydroxyurea	Reference	-
- Hydroxyurea	0.465 (0.138–1.562)	0.215

** Significant at *p* ≤ 0.05 level. Abbreviations: UOR, Unadjusted Odds Ratio; CI, confidence interval; ACS, Acute Chest Syndrome; SCD, Sickle Cell Disease; WBC, White Blood Cells; MCV, Mean Corpuscular Volume, RBC, Red Blood Cells; CRP, C-reactive Protein; NSAIDs, Non-steroidal Anti-inflammatory Drugs.

## Data Availability

The analyzed datasets used in this study and all analysis output reports are available upon reasonable request from the corresponding author. The data does not contain any identifiable data, and the confidentiality of the included patients is fully maintained. This is a sub-study of a descriptive retrospective study on pediatric sickle cell patients with acute chest syndrome that is currently in press [24]. Same dataset was utilized in both studies with different research question, objectives, analyses, and outcomes.

## Supplementary Materials

The following supporting information can be downloaded at: https://www.mdpi.com/article/10.3390/children9060894/s1, Table S1. Comparison between baseline hematological components in light of different SCD genotypes. Table S2. Laboratory data of the included patients throughout their admission stay. Table S3. Results of the receiver operating characteristic curve analyses for the predictive performance of the continuous variables toward recurrent ACS. Table S4. Predictive performance of the optimal cutoff values of the continuous variables for recurrent ACS group.

## Abbreviations

SCD: sickle cell disease; KSA, Kingdom of Saudi Arabia; ACS, Acute Chest Syndrome; VOC, Vaso-occlusive Crisis; LOS, Length of Stay; PICU, Pediatric Intensive Care Unit; KFHU, King Fahad Hospital of the University; IRB, Institutional Review Board; ER, Emergency Room; FDA, Food and Drug Administration; CRP, C-reactive Protein; SPSS, Statistic Package of Social Sciences; SD, Standard Deviation; IQR, Interquartile Range; UOR, Unadjusted Odds Ratio; CI, Confidence Interval; ROC, Receiver Operating Characteristics; AUC, Area Under Curve; AUROC, Area Under the Receiver Operating Characteristic Curve; PPV, Positive Predictive Value; NPV, Negative Predictive Value; LR+, Likelihood Ratio Positive; LR−, Likelihood Ratio Negative; WBC, White Blood Cells; MCV, Mean Corpuscular Volume; RBC, Red Blood Cells; URTI, Upper Respiratory Tract Infection; GI, Gastrointestinal; MCH, Mean Corpuscular Hemoglobin; NSAIDs, Nonsteroidal Anti-inflammatory Drugs.
