# Supplementary material for: Predictors of Recurrent Acute Chest Syndrome in Pediatric Sickle Cell Disease: A Retrospective Case-Control Study
Source: Children (Basel). 2022 Jun 15;9(6):894. doi: 10.3390/children9060894 (PMC9221711; doi:10.3390/children9060894)
Supplement: Supplementary file 1 [file children-09-00894-s001.zip › children-1692160-supplementary.pdf]

Table S1. Comparison between baseline hematological components in light of different SCD genotypes.

Study Variables	Both groups	Patients with a single ACS episode	Patients with recurrent ACS episodes	*p*-value
**Hemoglobin, mean ± SD**				
Total study population, [*n* = 31/42]	8.28 ± 0.88 g/dL	8.57 ± 0.9 g/dL	8 ± 0.80 g/dL	0.07[a]
Patients with Hgb SS subtype, [*n* = 23/29]	8.27 ± 0.74 g/dL	8.37 ± 0.79 g/dL	8.19 ± 0.73 g/dL	0.581[a]
Patients with Hgb Sβ0 thalassemia subtype, [*n* = 6/9]	8.28 ± 1.43 g/dL	9.4 ± 1.01 g/dL	7.17 ± 0.58 g/dL	0.03**[a]
Patients with Hgb Sα thalassemia subtype, [*n* = 2/4]	8.35 ± 1.06 g/dL	8.35 ± 1.06 g/dL	-	-
**MCV, mean ± SD**				
Total study population, [*n* = 31/42]	76.58 ± 13.11 fL	70.91 ± 15.47 fL	81.89 ± 7.64 fL	0.017**[a]
Patients with Hgb SS subtype, [*n* = 23/29]	78.1 ± 13.94 fL	71.72 ± 17.56 fL	83.02 ± 8.05 fL	0.052[a]
Patients with Hgb Sβ0 thalassemia subtype, [*n* = 6/9]	69.23 ± 8.71 fL	61.43 ± 1.45 fL	77.03 ± 2.24 fL	0.001**[a]
Patients with Hgb Sα thalassemia subtype, [*n* = 2/4]	81.1 ± 9.33 fL	81.1 ± 9.33 fL	-	-
**MCH, mean ± SD**				
Total study population, [*n* = 31/42]	30.13 ± 13.51 pg	29.32 ± 15.32 pg	30.89 ± 12.04 pg	0.751[a]
Patients with Hgb SS subtype, [*n* = 23/29]	30.03 ± 11.9 pg	32.51 ± 17.9 pg	28.13 ± 3.23 pg	0.395[a]
Patients with Hgb Sβ0 thalassemia subtype, [*n* = 6/9]	31.23 ± 21.59 pg	19.6 ± 0.66 pg	42.87 ± 27.55 pg	0.281[a]
Patients with Hgb Sα thalassemia subtype, [*n* = 2/4]	27.95 ± 3.46 pg	27.95 ± 3.46 pg	-	-
**RBC count, mean ± SD**				
Total study population, [*n* = 31/42]	3.2 ± 0.732 Mil/ul	3.52 ± 0.89 Mil/ul	2.91 ± 0.39 Mil/ul	0.024**[a]
Patients with Hgb SS subtype, [*n* = 23/29]	3.06 ± 0.51 Mil/ul	3.22 ± 0.61 Mil/ul	2.93 ± 0.4 Mil/ul	0.182[a]
Patients with Hgb Sβ0 thalassemia subtype, [*n* = 6/9]	3.83 ± 1.18 Mil/ul	4.84 ± 0.5 Mil/ul	2.81 ± 0.36 Mil/ul	0.005**[a]
Patients with Hgb Sα thalassemia subtype, [*n* = 2/4]	3.01 ± 0.69 Mil/ul	3.01 ± 0.69 Mil/ul	-	-
**Hematocrit, mean ± SD**				
Total study population, [*n* = 31/42]	24.58 ± 2.95 %	25.78 ± 3.48 %	23.45 ± 1.83 %	0.026**[a]
Patients with Hgb SS subtype, [*n* = 23/29]	24.34 ± 2.39 %	24.96 ± 3.06 %	23.85 ± 1.7 %	0.281[a]
Patients with Hgb Sβ0 thalassemia subtype, , [*n* = 6/9]	25.58 ± 4.85 %	29.47 ± 3.37 %	21.7 ± 1.47 %	0.022**[a]
Patients with Hgb Sα thalassemia subtype, [*n* = 2/4]	24.35 ± 3.32 %	24.35 ± 3.32 %	-	-
**Reticulocyte count, mean ± SD**				
Total study population, [*n* = 31/42]	9.57 ± 4.46 %	7.81 ± 3.95 %	11.21 ± 4.66 %	0.031**[a]
Patients with Hgb SS subtype, [*n* = 23/29]	9.76 ± 4.75 %	8.21 ± 3.95 %	10 ± 5.12 %	0.174[a]
Patients with Hgb Sβ0 thalassemia subtype, [*n* = 6/9]	8.63 ± 4.29 %	4.97 ± 1.38 %	12.3 ± 1.95 %	0.006**[a]
Patients with Hgb Sα thalassemia subtype, [*n* = 2/4]	10.1 ± 0.57 %	10.1 ± 0.57 %	-	-

[a] *p*-value was calculated using the independent Student's t-test. Abbreviation: ACS, Acute Chest Syndrome; SCD, Sickle Cell Disease; MCV, Mean Corpuscular Volume, MCH, Mean Corpuscular Hemoglobin; RBC, Red Blood Cells.

Table S2. Laboratory data of the included patients throughout their admission stay.

Study Variables	At admission	At ACS diagnosis	At PICU admission	24-hours prior to discharge
**WBC, median (IQR), [*n* = 91]**				
**Single, [*n* = 17]**	18.8 (10.2 - 25.45) k/ul<br>[*n* = 17]<br>P = 0.595[a]	17.7 (12.45 - 23.85) k/ul<br>[*n* = 17]<br>P = 0.720[a]	8.25 (7.46 - 9.03) k/ul<br>[*n* = 2]<br>P = 0.056[a]	8.15 (6.75 - 12.98) k/ul<br>[*n* = 6]<br>P = 0.188[a]
**Recurrent, [*n* = 74]**	19.6 (13.55 - 26.3) k/ul<br>[n = 73]<br>P = 0.595[a]	18.3 (13.55 - 24.65) k/ul<br>[*n* = 69]<br>P = 0.720[a]	29.1 (22.1 - 41.4) k/ul<br>[*n* = 7]<br>P = 0.056[a]	11.75 (8.28 - 15.35) k/ul<br>[*n* = 28]<br>P = 0.188[a]
**Neutrophil, mean ± SD, [*n* = 91]**				
**Single, [*n* = 17]**	66.67 ± 14.91 %<br>[*n* = 13]<br>P = 0.088[b]	68.64 ± 14.59 %<br>[*n* = 14]<br>P = 0.035**[b]	60.6 ± 20.65 %<br>[*n* = 2]<br>P = 0.238[b]	42.98 ± 20.96 %<br>[*n* = 4]<br>P = 0.850[b]
**Recurrent, [*n* = 74]**	58.01 ± 16.41 %<br>[*n*=53]<br>P = 0.088[b]	58.14 ± 16.49 %<br>[*n* = 50]<br>P = 0.035**[b]	77.06 ± 12.77 %<br>[*n* = 5]<br>P = 0.238[b]	41.21 ± 15.98 %<br>[*n* = 19]<br>P = 0.850[b]
**Eosinophils, median (IQR), [*n* = 91]**				
**Single, [*n* = 17]**	2 (1.4 - 3) %<br>[*n* = 11]<br>P = 0.582[a]	2 (0.08 - 3) %<br>[*n* = 10]<br>P = 0.854[a]	2.5 (2.25 - 2) %<br>[*n* = 2]<br>P = 1[a]	1.5 (1 - 7.25) %<br>[*n* = 4]<br>P = 0.113[a]
**Recurrent, [*n*=74]**	2 (1 - 3) %<br>[*n* = 51]<br>P = 0.582[a]	1.8 (1 - 3) %<br>[*n* = 46]<br>P = 0.854[a]	2 (1 - 3) %<br>[*n* = 2]<br>P = 1[a]	5 (2.85 - 8.5) %<br>[*n* = 21]<br>P = 0.113[b]
**Lymphocyte, mean + SD, [*n*=91]**				
**Single, [*n* = 17]**	21.91 ± 12.81 %<br>[*n* = 15]<br>P = 0.150[b]	21.69 ± 12 59 %<br>[*n* = 15]<br>P = 0.177[b]	33 ± 4.24 %<br>[*n* = 2]<br>P = 0.007**[b]	41.25 ± 18.68 %<br>[*n* = 4]<br>P = 0.978[b]
**Recurrent, [*n* = 74]**	27.30 ± 13.07 %<br>[*n* = 70]<br>P = 0.150[b]	26.88 ± 13.51 %<br>[*n* = 67]<br>P = 0.177[b]	13.1 ± 5.73 %<br>[*n* = 5]<br>P = 0.007**[b]	41 ± 16.77 %<br>[*n* = 21]<br>P = 0.978[b]
**RBC, mean ± SD, [*n* = 91]**				
**Single, [*n* = 17]**	3.08 ± 1.07 Mil/ul<br>[*n* = 17]<br>P = 0.342[b]	3.07 ± 0.04 Mil/ul<br>[*n* = 17]<br>P = 0.245[b]	0.67 ± 2.64 Mil/ul<br>[*n* = 2]<br>P = 0.918[b]	4.21 ± 0.32 Mil/ul<br>[*n* = 5]<br>P < 0.001**[b]
**Recurrent, [*n* = 74]**	2.82 ± 0.57 Mil/ul<br>[n = 72]<br>P = 0.342[b]	2.76 ± 0.53 Mil/ul<br>[n = 61]<br>P = 0.245[b]	2.91 ± 0.64 Mil/ul [n = 7]<br>P = 0.918[b]	3.31 ± 0.5 Mil/ul<br>[n = 27]<br>P < 0.001**[b]
**Hemoglobin, mean ± SD, [*n* = 91]**				
**Single, [*n* = 17]**	7.53 ± 2.05 g/dL<br>[*n* = 17]<br>P = 0.946[b]	7.49 ± 2.03 g/dL<br>[*n* = 17]<br>P = 0.964[b]	5.75 ± 4.74 g/dL<br>[*n* = 2]<br>P = 0.611[b]	9.82 ± 0.09 g/dL<br>[*n* = 6]<br>P = 0.127[b]
**Recurrent, [*n* = 74]**	7.57 ± 1.18 g/dL<br>[*n* = 71]<br>P = 0.946[b]	7.5 ± 1.23 g/dL<br>[*n* = 67]<br>P = 0.964[b]	8.09 ± 1.89 g/dL<br>[*n* = 7]<br>P = 0.611[b]	9.17 ± 0.88 g/dL<br>[*n* = 29]<br>P = 0.127[b]
**Hematocrit, mean ± SD, [*n* = 91]**				
**Single, [*n* = 17]**	22.44 ± 6.07 %<br>[*n* = 17]<br>P = 0.768[b]	22.36 ± 5.99 %<br>[*n* = 17]<br>P = 0.779[b]	18 ± 14.43 %<br>[*n* = 2]<br>P = 0.646[b]	30.6 ± 2.77 %<br>[*n* = 5]<br>P = 0.14[b]
**Recurrent, [*n* = 74]**	22.90 ± 3.79 %<br>[*n* = 72]<br>P = 0.768[b]	22.77 ± 3.87 %<br>[*n* = 69]<br>P = 0.779[b]	24.33 ± 5.74 %<br>[*n* = 7]<br>P = 0.646[b]	27.14 ± 2.74 %<br>[*n* = 28]<br>P = 0.14[b]
**Reticulocyte, median (IQR), [*n* = 91]**				
**Single, [*n* = 17]**	8.9 (5.23 - 12.25) %<br>[*n* = 16]<br>P = 0.301[a]	7.9 (3.5 - 13.1) %<br>[*n* = 15]<br>P = 0.407[a]	12 (6.75 - 17.25) %<br>[*n* = 2]<br>P = 1[a]	5.25 (1.28 - 6.15) %<br>[*n* = 6]<br>P = 0.850[a]
**Recurrent, [*n* = 74]**	9.6 (7 - 12.8) %<br>[*n* = 67]<br>P = 0.301[a]	8.7 (6.38 - 12.4) %<br>[*n* = 62]<br>P = 0.407[a]	6.3 (3.95 - 13.35) %<br>[*n* = 5]<br>P = 1[a]	3.3 (2 - 12) %<br>[*n* = 15]<br>P = 0.850[a]
**MCV, mean ± SD, [*n* = 91]**				
**Single, [*n* = 17]**	75.07 ± 10.14 fL [*n* = 17]<br>P = 0.006**[b]	74.93 ± 9.54 fL<br>[*n* = 17]<br>P = 0.007**[b]	73.65 ± 16.19<br>[*n* = 2]<br>P = 0.303[b]	73.3 ± 10.40 fL<br>[*n* = 5]<br>P = 0.026**[b]
**Recurrent, [*n* = 74]**	82.25 ± 9.24 fL<br>[*n* = 72]	81.86 ± 9.12 fL<br>[*n* = 69]	83.97 ± 10.62<br>[*n* = 7]	82.89 ± 8.07 fL<br>[*n* = 27]

	P = 0.006**[b]	P = 0.007**[b]	P = 0.303[b]	P = 0.026**[b]
**MCH, mean ± SD, [*n* = 91]**				
**Single, [*n* = 17]**	25.41 ± 4 pg [*n* = 17] P = 0.033**[b]	25.25 ± 3.87 pg [*n* = 17] P = 0.034**[b]	22.93 ± 5.70 pg [*n* = 3] P = 0.093[b]	25.23 ± 2.84 pg [*n* = 4] P = 0.111[b]
**Recurrent, [*n* = 74]**	27.55 ± 3.56 pg [*n* = 72] P = 0.033**[b]	27.32 ± 3.47 pg [*n* = 69] P = 0.034**[b]	28.86 ± 4.03 pg [*n* = 7] P = 0.093[b]	27.87 ± 3.03 pg [*n* = 27] P = 0.111[b]
**Platelets, mean ± SD, [*n* = 91]**				
**Single, [*n* = 17]**	337.47 ± 181.62 k/ul [*n* = 17] P = 0.396[b]	343.12 ± 179.47 k/ul [*n* = 17] P = 0.571[b]	155 ± 36.77 [*n* = 2] P = 0.097[b]	491 ± 312.96 k/ul [*n* = 6] P = 0.719[b]
**Recurrent, [*n* = 74]**	377.48 ± 171.61 k/ul [*n* = 71] P = 0.396[b]	360.81 ± 171.46 k/ul [*n* = 68] P = 0.571[b]	356.57 ± 140.74 [*n* = 7] P = 0.097[b]	543.43 ± 321.85 k/ul [*n* = 28] P = 0.719[b]
**Total bilirubin, median (IQR), [*n* = 91]**				
**Single, [*n* = 17]**	3.1 (2.1 - 3.45) mg/dL [*n* = 8] P = 0.839[a]	2.7 (1.4 - 3.45) mg/dL [*n* = 8] P = 0.571[a]	2.1 (1.5 - 2.7) mg/dL [*n* = 2] P = 1[a]	0 [*n* = 0] P = -
**Recurrent, [*n* = 74]**	2.6 (2.03 - 3.85) mg/dL [*n* = 40] P = 0.839[a]	2.6 (2.6 - 3.8) mg/dL [*n* = 35] P = 0.571[a]	2.5 (2 - 2.75) mg/dL [*n* = 3] P = 1[a]	1.45 (0.73 - 3.38) mg/dL [*n* = 4] P = -
**Direct bilirubin, median (IQR), [*n* = 91]**				
**Single, [*n* = 17]**	0.55 (0.38 - 0.88) mg/dL [*n* = 8] P = 0.792[a]	0.53 (0.38 - 0.6) mg/dL [*n* = 8] P = 0.846[a]	0.43 (0.37 - 0.50) mg/dL [*n* = 2] P = 1[a]	0 [*n* = 0] P = -
**Recurrent, [*n* = 74]**	0.5 (0.33 - 0.7) mg/dL [*n* = 39] P = 0.792[a]	0.5 (0.32 - 0.7) mg/dL [*n* = 36] P = 0.846[a]	0.46 (0.39 - 0.53) mg/dL [*n* = 2] P = 1[a]	0.33 (0.23 - 0.49) mg/dL [*n* = 4] P = -
**LDH, mean ± SD, [*n* = 91]**				
**Single, [*n* = 17]**	519.63 ± 169.32 U/L [*n* = 8] P = 0.662[b]	515.5 ± 181.64 U/L [*n* = 8] P = 0.523[b]	415 ± 212.13 U/L [*n* = 2] P = 0.331[b]	0 [*n* = 0] P = -
**Recurrent, [*n* = 74]**	548.29 ± 166.87 U/L [*n* = 38] P = 0.662[b]	563.17 ± 190.20 U/L [*n* = 35] P = 0.523[b]	755.33 ± 364.71 U/L [*n* = 3] P = 0.331[b]	480.75 ± 217.61 U/L [*n* = 4] P = -
**Alkaline phosphatase, mean ± SD [*n* = 91]**				
**Single, [*n* = 17]**	141.38 ± 52.47 U/L [*n* = 8] P = 0.392[b]	128.75 ± 50.17 U/L [*n* = 8] P = 0.210[b]	117.50 ± 72.832 U/L [*n* = 2] P = 0.866[b]	0 [*n* = 0] P = -
**Recurrent, [*n* = 74]**	156.78 ± 44.29 U/L [*n* = 37] P = 0.392[b]	150.82 ± 42.49 U/L [*n* = 33] P = 0.210[b]	106.50 ± 12.02 U/L [*n* = 2] P = 0.866[c]	132.33 ± 58.23 U/L [*n* = 3] P = -
**Positive CRP-Qualitative, *n* (%) [*n* = 91]**				
**Single, [*n* = 17]**	10 (66.7%) [*n* = 15] P = 0.007**[c]	12 (92.3%) [*n* = 13] P = 0.460[c]	2 (100%) [*n* = 2] P = 1[c]	2 (100%) [*n* = 2] P = -
**Recurrent, [*n* = 74]**	56 (94.9%) [*n* = 59] P = 0.007**[c]	56 (96.6%) [*n* = 58] P = 0.460[c]	4 (80%) [*n* = 5] P = 1[c]	5 (100%) [*n* = 5] P = -
**ESR, mean ± SD, [*n* = 91]**				
**Single, [*n* = 17]**	27.42 ± 19.53 mm/hr [*n* = 12] P = 0.880[b]	47 ± 33.61 mm/hr [*n* = 12] P = 0.094[b]	52 ± 0 mm/hr [*n* = 1] P = 0.616[b]	81 ± 55.15 mm/hr [*n* = 2] P = 0.771[b]
**Recurrent, [*n* = 74]**	28.32 ± 18.21 mm/hr [*n* = 49] P = 0.880[b]	28.88 ± 17.61 mm/hr [*n* = 53] P = 0.094[b]	37 ± 22.11 mm/hr [*n* = 3] P = 0.616[b]	66.29 ± 20.28 mm/hr [*n* = 7] P = 0.771[b]

* Missing details were excluded. ** Significant at p ≤ 0.05 level. [a] P-value was calculated using the Mann Whitney U test; [b] P-value was calculated using the independent t-test; [c] P-value was calculated using a chi-square test or fisher-exact test. Abbreviations: ACS, Acute Chest Syndrome; PICU, Pediatric Intensive Care Unit; WBC, White Blood Cells; IQR, Interquartile range; SD, Standard Deviation; RBC, Red Blood Cells; MCV, Mean Corpuscular Volume; MCH, Mean Corpuscular Hemoglobin; LDH, Lactate Dehydrogenase; CRP; C-reactive Protein; ESR, Erythrocyte Sedimentation Rate.

Table S3. Results of the Receiver Operating Characteristic Curve analyses for the predictive performance of the continuous variables toward recurrent ACS.

Study Variables	Test Direction	AUROC (95% CI)	*p*-value
**Age at time of first ACS diagnosis, [*n* = 42/42]**	Smaller value indicates higher probability of recurrent ACS.	0.833 (0.702-0.964)	< 0.001**
**Age at time of all ACS episodes' diagnoses, [*n* = 91/91]**	Smaller value indicates higher probability of recurrent ACS.	0.7 (0.574-0.825)	0.011**
**SCD-related hospitalizations/year, [*n* = 37/42]**	Larger value indicates higher probability of recurrent ACS.	0.72 (0.558-0.883)	0.026**
**Baseline WBC count, [*n* = 29/42]**	Larger value indicates higher probability of recurrent ACS.	0.786 (0.621-0.95)	0.009**
**Baseline MCV, [*n* = 31/42]**	Larger value indicates higher probability of recurrent ACS.	0.767 (0.588-0.946)	0.011**
**Baseline RBC count, [*n* = 31/42]**	Smaller value indicates higher probability of recurrent ACS.	0.7 (0.504-0.896)	0.058
**Baseline hematocrit, [*n* =31/42]**	Smaller value indicates higher probability of recurrent ACS.	0.754 (0.563-0.946)	0.016**
**Baseline reticulocyte count, [*n* =31/42]**	Larger value indicates higher probability of recurrent ACS.	0.721 (0.539-0.903)	0.036**
**Neutrophil at time of ACS diagnosis, [*n* = 64/91]**	Smaller value indicates higher probability of recurrent ACS.	0.686 (0.536-0.837)	0.034**
**RBC count 24 hours before discharge, [*n* = 32/91]**	Smaller value indicates higher probability of recurrent ACS.	0.926 (0.833-1)	0.003**
**MCV at time of admission, [*n* = 89/91]**	Larger value indicates higher probability of recurrent ACS.	0.701 (0.556-0.845)	0.01**
**MCV at time of diagnosis, [*n* = 86/91]**	Larger value indicates higher probability of recurrent ACS.	0.709 (0.562-0.856)	0.008**
**MCV 24 hours before discharge, [*n* = 32/91]**	Larger value indicates higher probability of recurrent ACS.	0.785 (0.551-1)	0.046**

** Significant at $p \leq 0.05$ level. Abbreviations: AUROC, area under the receiver operating characteristic curve; CI, confidence interval; ACS, Acute Chest Syndrome; SCD, Sickle Cell Disease; WBC, White Blood Cells; MCV, Mean Corpuscular Volume, RBC, Red Blood Cells.

Table S4. Predictive performance of the optimal cutoff values of the continuous variables for recurrent ACS group.

Study Variables	Cutoff value	Sensitivity %	Specificity %	PPV %	NPV %	LR+	LR-
**Age at time of first ACS diagnosis, [*n* = 42/42]**	≤7.5	84	76.5	84	76.5	3.57	0.21
**Years from SCD diagnosis to the first ACS episode, [*n* = 37/42]**	≤4.75	65.2	78.6	83.3	57.9	3.05	0.44
**Age at time of all ACS episodes' diagnoses, [*n* = 91/91]**	≤7.5	59.5	76.5	91.7	30.2	2.53	0.53
**SCD-related hospitalizations/year, [*n* = 37/42]**	≥2.5	73.9	57.1	73.9	57.1	1.72	0.46
**Baseline WBC count, [*n* = 29/42]**	≥11.85	73.3	64.3	68.8	69.2	2.05	0.42
**Baseline MCV, [*n* = 31/42]**	≥76.25	81.3	73.3	76.5	78.6	3.04	0.26
**Baseline RBC count, [*n* = 31/42]**	≤3.255	87.5	60	70	81.8	2.19	0.21
**Baseline hematocrit, [*n* = 31/42]**	≤24.6	75	73.3	75	73.3	2.81	0.34
**Baseline reticulocyte count, [*n* = 31/42]**	≥8.6	68.8	60	64.7	64.3	1.72	0.52
**Neutrophil at time of ACS diagnosis, [*n* = 64/91]**	≤61.3	58	78.6	90.6	34.4	2.71	0.53
**RBC count 24 hours before discharge, [*n* = 32/91]**	≤3.855	88.9	100	100	62.5	-	0.111
**MCV at time of admission, [*n* = 89/91]**	≥75.55	80.6	58.8	89.2	41.7	1.96	0.33
**MCV at time of diagnosis, [*n* = 86/91]**	≥75.55	79.7	64.7	90.2	44	2.26	0.31
**MCV 24 hours before discharge, [*n* = 32/91]**	≥80.95	66.7	80	94.7	30.8	3.34	0.42
**Positive CRP at time of admission, [*n* = 74/91]**	-	94.9	33.3	84.8	62.5	1.42	0.15

Abbreviation: PPV, Positive Predictive Value; NPV, Negative Predictive Value; LR+, Likelihood Ratio Positive; LR-, Likelihood Ratio Negative; ACS, Acute Chest Syndrome; SCD, Sickle Cell Disease; WBC, White Blood Cells; MCV, Mean Corpuscular Volume, RBC, Red Blood Cells; CRP, C-reactive Protein.
